# Synthesis of Monolayer Gold Nanorings Sandwich Film and Its Higher Surface-Enhanced Raman Scattering Intensity

**DOI:** 10.3390/nano10030519

**Published:** 2020-03-13

**Authors:** Liqiu Zhang, Tiying Zhu, Cheng Yang, Ho Young Jang, Hee-Jeong Jang, Lichun Liu, Sungho Park

**Affiliations:** 1 Department of Chemistry & Department of Energy Science, Sungkyunkwan University, Suwon 440-746, Korea; zeroair@skku.edu (H.Y.J.); snow306@skku.edu (H.-J.J.); 2 College of Biological, Chemical Sciences and Engineering & Nanotechnology Research Institute, Jiaxing University, Jiaxing 314001, China; lichun.liu@zjxu.edu.cn; 3 School of Physics and Electronics, Shandong Normal University, Jinan 250014, China; 2018020534@stu.sdnu.edu.cn (T.Z.); chengyang@sdnu.edu.cn (C.Y.)

**Keywords:** SERS, sandwich, Au nanoring, Ag mirror, Ag cover, electromagnetic field

## Abstract

Most previous studies relating to surface-enhanced Raman spectroscopy (SERS) signal enhancement were focused on the interaction between the light and the substrate in the *x*-*y* axis. 3D SERS substrates reported in the most of previous papers could contribute partial SERS enhancement via *z* axis, but the increases of the surface area were the main target for those reports. However, the *z* axis is also useful in achieving improved SERS intensity. In this work, hot spots along the *z* axis were specifically created in a sandwich nanofilm. Sandwich nanofilms were prepared with self-assembly and Langmuir-Blodgett techniques, and comprised of monolayer Au nanorings sandwiched between bottom Ag mirror and top Ag cover films. Monolayer Au nanorings were formed by self-assembly at the interface of water and hexane, followed by Langmuir-Blodgett transfer to a substrate with sputtered Ag mirror film. Their hollow property allows the light transmitted through a cover film. The use of a Ag cover layer of tens nanometers in thickness was critical, which allowed light access to the middle Au nanorings and the bottom Ag mirror, resulting in more plasmonic resonance and coupling along perpendicular interfaces (*z*-axis). The as-designed sandwich nanofilms could achieve an overall ~8 times SERS signals amplification compared to only the Au nanorings layer, which was principally attributed to enhanced electromagnetic fields along the created *z*-axis. Theoretical simulations based on finite-difference time-domain (FDTD) method showed consistent results with the experimental ones. This study points out a new direction to enhance the SERS intensity by involving more hot spots in *z*-axis in a designer nanostructure for high-performance molecular recognition and detection.

## 1. Introduction

Surface-enhanced Raman spectroscopy (SERS) is a surface-sensitive technique that amplifies the Raman scattering intensities through molecules adsorbed on rough metal surfaces or nanostructured films subjected to light irradiation [1,2,3,4,5,6,7]. Several advantages of SERS like high sensitivity, free of labelling and instant chemicals fingerprinting have made it useful in many application fields, such as food, biology, medicine, chemistry, and environment [8,9,10,11,12]. Hence, intensive experimental and theoretical studies have recently been directed towards improving SERS signal intensities. It is widely recognized that composition, size, shape of nanostructures, types of surface molecules, and wavelength of incident light are strongly correlated to SERS signal intensity. The origin of SERS signal comes from the enhancement in electromagnetic field provided by plasmonic surface molecules when incident light strikes the substrate surface. This mechanism has been widely acknowledged as the electromagnetic (EM) theory [13].

The EM theory suggests that the electromagnetic field becomes significantly enhanced at edges and interfacial gaps between nanostructures due to strong out-plane plasmonic coupling, or so-called ‘hot spot’ [9,10]. So far, numerous published reports have addressed highly sensitive SERS substrates through the increase in density of interfacial hot spots among various nanostructures in *x*-*y* planes. In particular, Au and Ag nanostructures, such as nanospheres [14,15,16], nanoprisms [17,18,19], nanoplates [20,21,22,23], nanocubes [24,25,26], nanorings [27,28,29], and nanostars [30,31,32,33] have been extensively studied as SERS substrates due to their active surface plasmon resonance, though aluminum (Al) was recently recognized as plasmonic metal and SERS substrate [34]. In addition, the strategy of constructing 3D structure of plasmonic Au or Ag has been adopted to increase effective surface area of SERS substrate [35,36,37,38]. Surface plasmon coupling in nanostructured films is usually strong along the *x*-*y* directions since incident light can simultaneously strike the adjacent nanostructures along the *x*-*y* plane. Most previous works dealt with this type of hot spots in horizontal plane [39,40,41,42]. However, plasmonic coupling along third dimension issued directly from the *z*-axis (vertical direction) is another source of hot spots that enables to enhance SERS intensity [43,44,45]. SERS signal amplification via constructing more active sites from the *z*-axis is scarcely reported and requires scientific exploration.

Ultrahigh enhancement in electromagnetic fields has been determined by numerical simulations and experimental testing using Au nanospheres deposited on Ag mirror as a model [46]. The use of an Ag mirror predominated the enhancement of the electromagnetic field along the *z*-axis. By evaluating the plasmon energy as a function of different distances between two Au nano-octahedrons and vectors of incident light waves, Yang et al. [43] found “dark modes” plasmons that are stronger along the *z*-axis when compared to parallel *x*-*y* axis. In addition, a *z*-axis-polarized oscillating dipole could be produced at the junctions between two adjacent Au nanomesh layers. The electron oscillation in one mesh could generate the oscillated image charge in the neighboring nanomesh, as demonstrated by Jung et al. [47]. Inspired by these theoretical and experimental findings, we rationally designed a novel 3D sandwich nanofilm to enhance the density of hot spots along the *z*-axis. The sandwich nanofilm based on monolayer Au nanorings sandwiched between Ag mirror and Ag cover was successfully synthesized and the SERS intensity enhancement was successfully observed. This investigation provides a new strategy to enhance SERS intensity from the *z*-axis in nanostructured SERS substrates.

## 2. Experimental Section

### 2.1. Materials

Chloroplatinic acid (H_2_PtCl_6_·6H_2_O), chloroauric acid (HAuCl_4_·3H_2_O), and silver nitrate (AgNO_3_), Au, Ag, Ti powder, Si wafer were purchased from Aldrich (Louis, MO, USA). Sodium citrate (Na_3_C_6_H_5_O_7_·2H_2_O), CTAB (C_16_H_33_(CH_3_)_3_NBr), potassium iodide (KI), ascobic acid (C_6_H_8_O_6_), poly(vinyl alcohol) (PVA), polymethyl methacrylate (PMMA), (3-mercaptopropyl) trimethoxysilane (MPTMS), hydrochloric acid (HCl), ethanol (C_2_H_5_OH), and hexane (C_6_H_6_) were purchased from Daejeong Chemical Inc. (SIHEUNG, Korea). Anodic aluminum oxide membranes (AAO, 13 mm diameter, ~200 nm pore size, 60 μm thickness) were purchased from Whatman International (Dassel, Germany). All aqueous solutions were prepared by fresh deionized water (18.2 MΩ·cm).

### 2.2. Synthesis of Au Nanorings

The synthetic pathway for Au nanorings with Pt skeleton followed the experimental procedure in our previous publication [48] with a minor modification. In this synthetic process, we synthesized Au nanoprisms, Au nanoplates, Au@Pt nanoplates, and Pt@Au nanorings, respectively. Au nanoprisms were synthesized from 5 nm spherical seeds by a three-step, seed-mediated method as reported previously [49]. Au nanoplates were grown from Au nanoprisms [50]. All of the Au nanoplate solutions were optically normalized to the same effective concentration. The synthetic pathway for Au@Pt nanoplates (rim-preferential growth of Pt on Au nanoplates) followed the experimental procedure in our previous paper [51]. To a 50 mL-vial was added 20 mL of 0.05 M CTAB (with iodide ions, 50 μM)), 5 mL of redispersed Au nanoplates, 30 μL of 2 mM aqueous AgNO_3_ solution, and 480 μL of 0.1 M aqueous ascorbic acid solution, sequentially. The mixture was kept at 70 °C. After 1 h, 480 μL of 0.1 M HCl, and 300 μL of 2 mM aqueous H_2_PtCl_6_ solution were added to the mixture with gentle shaking. The mixture was kept at 70 °C for approximately 4 h. After this reaction, the sample was washed twice by centrifugation and then dispersed into 15 mL of DI water for preparation of the stock solution. For the preparation of Pt@Au nanorings, Au moiety in Au@Pt nanoplates was selectively etched away by the following recipe. To etch the central Au section of the nanoplates, 40 mL of 0.05 M CTAB with iodide ions (50 μM), and 150 μL of 20 mM aqueous HAuCl_4_ solution were added to the vial containing 8 mL Au@Pt nanoplates. The mixture was kept at 50 °C for 30 min. Next, 380 μL of 0.1 M aqueous ascorbic acid solution was added to the mixture, which induced the regrowth of Au on the Pt nanorings. The mixture was kept at 30 °C for 4 h. After this reaction, the sample was centrifuged, and the supernatant was removed and redispersed in DI water. After washing twice, Pt@Au nanorings were dispersed into 10 mL of DI water for assembling a two-dimensional monolayer of nanorings. The outer and inner diameters of the nanorings were 129 ± 11 and 45 ± 6 nm, respectively. The final uniform product was Au-coated Pt nanorings, abbreviated as Au nanorings consistently.

### 2.3. Langmuir-Blodgett Monolayer of Au Nanorings

Two-dimensional arrays of nanorings were prepared at a water/hexane interface by entrapping nanorings following the published works [52,53]. The driving force for entrapment is the decrease of the interfacial energy between water and hexane. 20 mL of the Au nanorings aqueous solution was transferred to a Teflon cell (inner dimension, 7.5 × 4.0 × 2.0 cm) filled with water which was connected to a circulator to control the temperature of the colloid solution to 5 °C. 5 mL of hexane was added to the top of the colloid solution to form an immiscible hexane/water interface and 1 mL 1 mM of MPTMS ((3-mercaptopropyl) trimethoxysilane) was added to the hexane layer. Then, about 8 mL of ethanol was added dropwise at a rate of 0.5 mL/min using a mechanical syringe pump, to extract Au nanorings from the aqueous solution to the hexane/water interface. The detailed process to fabricate the 2-dimensional array of nanorings films can also be found in a previous publication [54]. The nanorings films were transferred onto Si wafer or Si/Ti/Au or Si/Ti/Ag support substrate for FESEM and SERS measurements.

### 2.4. Fabrication of Si/Ti/Au(Ag) Support Substrate

The junction between the silicon wafer and Au(Ag) mirror was so weak that it was easy to peel off. Thus thin Ti film was deposited on the silicon wafer before making Au(Ag) mirror to ensure a strong adherence, forming Si/Ti/Au(Ag) wafer. E-beam deposition method was used to deposit thin Ti film (~15 nm at 0.01–0.1 nm/s) on a clean silicon wafer which was pretreated with sonication in acetone and ethanol for 10 min respectively and immersed in ethanol before use. Then, Au (Ag) mirrors were deposited on Ti film using a sputter coater (Cressington 108 auto) for 300 s at a current level of 30 mA. Their thickness of Au or Ag is fixed at ~200 nm. The Si/Ti/Au(Ag) (abbreviated as Au or Ag mirror) was used as a substrate to collect the floating monolayer of Au nanorings at the interface in the solution.

### 2.5. Fabrication of Ag Cover

AAO/PVA/Ag/PMMA structure was made as followed: firstly, PVA (Poly(vinyl alcohol)) film was formed on the back side of the commercial AAO by spin coating at 3500 rpm for 1 min. Secondly, thin Ag layers were then deposited on the AAO/PVA film by e-beam deposition method through controlling the deposition speed, such as 0.01–0.05 nm/s, 0.01–0.1 nm/s, 0.1–0.3 nm/s for ~9, 15, 30 nm Ag films, respectively. Their thicknesses were evaluated by an atomic force microscopy (AFM). Lastly, poly (methyl methacrylate) (PMMA) was coated onto the AAO/PVA/Ag layer forming AAO/PVA/Ag/PMMA through the spin coating at 3500 rpm for 1 min using a PMMA dissolved in dichlorobenzene solution (MICRO CHEM 950 PMMA C 4). In order to form the Ag cover, we put the AAO/PVA/Ag/PMMA film into 3M NaOH to dissolve AAO and PVA resulting in Ag/PMMA film floating on the top of the NaOH solution. Deionized water was used to replace the NaOH for getting the clean Ag/PMMA film which was transferred to the monolayer nanorings substrate resulting in the final SERS substrate and dried in an oven at 80 °C. To obtain the pure sandwich nanofilm, the PMMA layer was dissolved by immersion in acetone for 30 min. The existence of Ag cover was confirmed by SEM and EDS data as shown in Figure S1.

### 2.6. SERS Measurements

A confocal Raman spectrometer (WiTec alpha 300R) was used to record the SERS signal intensity. A He-Ne laser (632.8 nm) was used for the excitation source. All of the samples were dipped into 0.1 M benzenethiol in an ethanol solution for 1 h for molecules staining. The SERS measurements for three samples in each set were usually reproduced three times to consolidate the accuracy of the measurement using average intensity of signal intensity at 1573 cm^−1^.

### 2.7. Characterizations

Field-emission scanning electron microscopy (FESEM; JEOL 7600F, Akishima, Tokyo, Japan) was used to observe structural properties of the Au nanoring structures. The transmittance of the Ag covers immobilized on the glass slide was obtained by a UV-visible (UV-vis) spectrometer (Scinco S-3100, Scinco, Seoul, Korea). Thicknesses of Ag on Si wafer and the wire diameter of nanorings were measured by atomic force microscopy (AFM) (Park systems NX 10, Park Systems Corp., Suwon, Korea). A COMSOL software (5.0, COMSOL Co., Ltd., Burlington, VT, USA) was used to conduct the Finite-Difference Time-Domain (FDTD) simulations.

## 3. Results and Discussion

**Figure 1:**
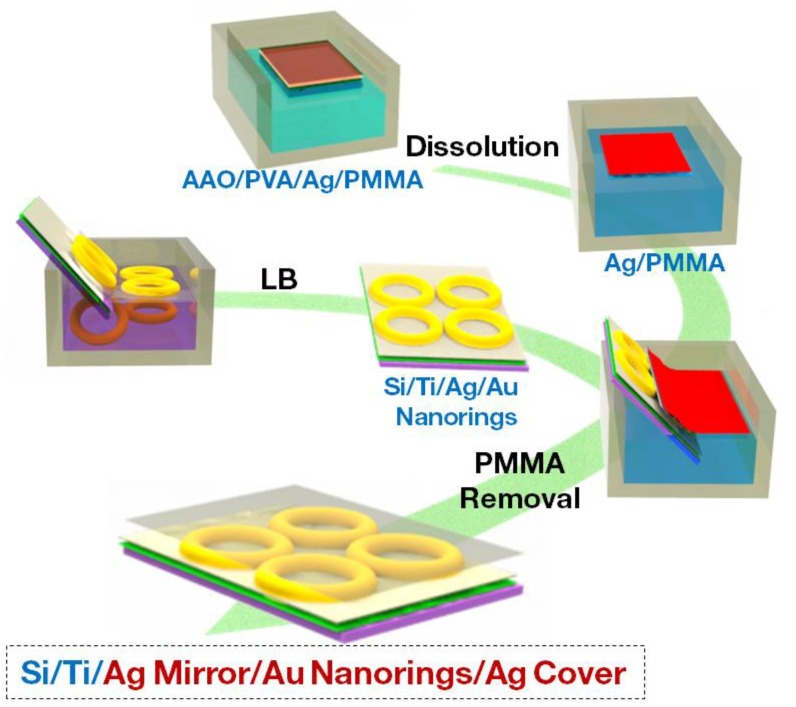
Flow chart of fabrication process of Ag mirror/Au nanorings/Ag cover sandwich nanofilm. The mirror refers to substrate underlying the layer of Au nanorings, and the cover represents top layer on Au nanorings. LB denotes Langmuir-Blodgett technique.

The synthetic route for the as-proposed 3D sandwich nanofilm of Ag mirror/Au nanorings/Ag cover is illustrated in Figure 1. The size of Au nanorings was ~130 nm in outer diameter and ~45 nm in inner diameter. The general fabrication process of the sandwich nanofilm was based on the Langmuir-Blodgett (LB) technique. The production of tightly packed 2D monolayers of Au nanorings was first investigated, and highlighted the importance of MPTMS ((3-mercaptopropyl) trimethoxysilane) in the solution during Au nanorings self-assembly into monolayer. Au nanorings entrapped at the interface between water and hexane possessed residual surface charges with electrostatic repulsions vis-a-vis of neighboring Au nanorings. However, addition of MPTMS into the hexane layer led to adsorption of thiol end functional group onto the Au nanorings surface through condensation and dehydration, [45] resulting in formation of an MPTMS self-assembled monolayer on Au nanorings surfaces. Thus, the concentration of MPTMS affected the degree of close packing. In Figure S2, Au nanorings deposited on substrates looked relatively well arranged when 1 mL 1 mM MPTMS was added to the hexane layer. By contrast, excess amounts of MPTMS can cause severe aggregation, as depicted in Figure S2C.

In UV-vis-NIR spectroscopy, two LSPR (localized surface plasmon resonance) bands attributed to Au nanorings film emerged at 514 and 779 nm (Figure S3), corresponding to out-plane and in-plane dipole resonance modes, respectively. The wide extinction band at 779 nm led to enhanced electromagnetic field at the excitation wavelength. The Stokes-Raman mode improved SERS performance of the plasmonic nanostructures. Because only 633 nm was used as the excitation wavelength, the attention was focused on the second resonance peak. Although the excitation wavelength was off resonance using 633 nm excitation, the emission wavelength of Stokes-Raman signals of benzenethiol from 675 nm to 702 nm matched well with the second plasmonic resonance, leading to enhanced Raman emission (Equation (S1)).

**Figure 2:**
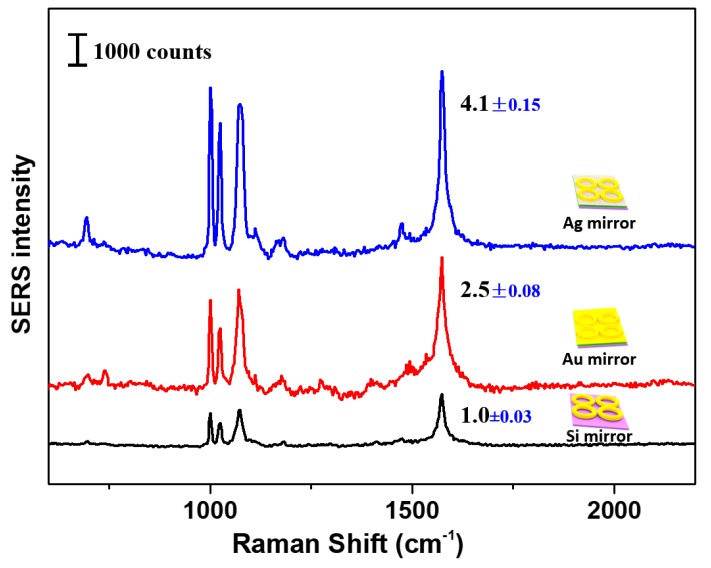
SERS intensities of benzenethiol molecules at monolayered Au nanorings deposited on Si (black trace), Au (red trace), and Ag (blue trace) mirrored substrates.

**Figure 3:**
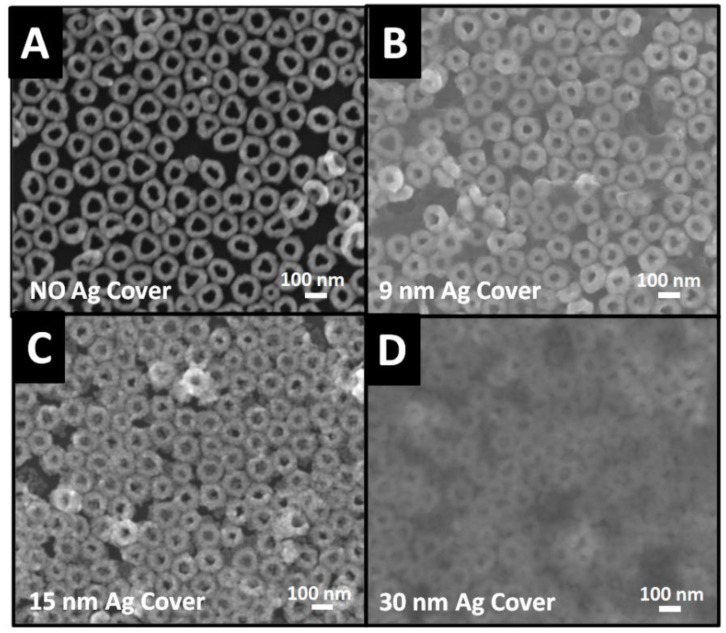
SEM images of Ag covers deposited on monolayered Au nanorings with different thickness: (**A**) 0 nm, (**B**) 9 nm, (**C**) 15 nm, and (**D**) 30 nm. The surface morphology of Ag cover may be affected during SEM measurements.

Figure 2 shows the SERS spectra of monolayered Au nanorings deposited on substrates with different mirrors. The assignment of peaks present in SERS spectra has previously been carried out, [55,56] and will not be repeated here since this report aimed to study SERS enhancement brought by mirror and cover effects. As shown in Figure 2, Au nanorings deposited on support substrates with Au and Ag mirrors illustrated largely enhanced SERS signal intensities when compared to those prepared without mirrors, such as Au nanorings deposited on pure Si substrates (Figure 3A). The SERS signals of Au nanorings deposited on Si substrate originated from the hot spots present in gaps between Au nanorings, providing enhanced electromagnetic fields. Under such circumstances, SPR coupling in monolayer Au nanorings was only effective along the *x* or *y* plane [57]. Thus, enhancement of the electromagnetic field due to SPR coupling was absent along the *z*-axis plane. By contrast, SERS activities were significantly improved on both Au and Ag mirrored substrates when compared to those made of only Si substrate, since both Au and Ag are plasmonic metals enable of producing substantial plasmonic coupling along the *z*-axis between nanoring and Au or Ag on the substrate. In particular, Au nanorings deposited on Ag mirror showed the strongest SERS response among all tested specimens. It is needed to notice that negligible SERS was observed for the pure Ag film comparing with the case of Au nanorings on the Ag film (Figure S4). Previous findings suggested that *z*-axis heterogeneous junctions between Au and Ag nanomeshes could induce stronger electromagnetic fields when compared to Au-Au and Ag-Ag nanomeshes [47]. The differences in electronegativity between the two metals more readily produced image charges at the counterpart when compared to homogeneous junctions. As a result, Au nanorings deposited on Ag mirror film also experimentally exhibited the higher SERS activities than Au nanorings on Au mirror films (Figure 2).

To further study the image charge effect, Ag nanofilm was placed on Ag film mirrored monolayer Au nanorings as a cover to yield Ag cover/Au nanorings/Ag mirror structure. The cover was expected to have a similar role as that of a mirror. The direct deposition of thin flat cover on hollow Au nanorings surface was quite challenging. Hence, an immobilization strategy was adopted, where flat Ag nanofilms were first deposited by e-beam technique using PVA modified AAO membrane at controlled thickness of 9 nm (9.3 ± 2.3), 15 nm (14.8 ± 2.9), and 30 nm (30.1 ± 6.2). The details were provided in the Section 2 and thickness was evaluated by AFM measurements. On the other hand, thickness of Ag cover was critical in SERS intensity since thicker covers could partially or entirely block the penetration of incident light. The thin Ag cover with several nanometers appeared almost transparent under SEM (Figure 3B), allowing light to penetrate inside the film and cause electron-coupling at both Au nanorings-Ag mirror and Au nanorings-Ag cover. By comparison, the Au nanorings beneath 30 nm-Ag cover became blurry due to large thickness (Figure 3D). At Ag cover thickness less than 30 nm, Au nanorings became visible as indicated by SEM (Figure 3A–C). The little packing disorder of Au nanorings was caused by sampling during SEM measurements.

**Figure 4:**
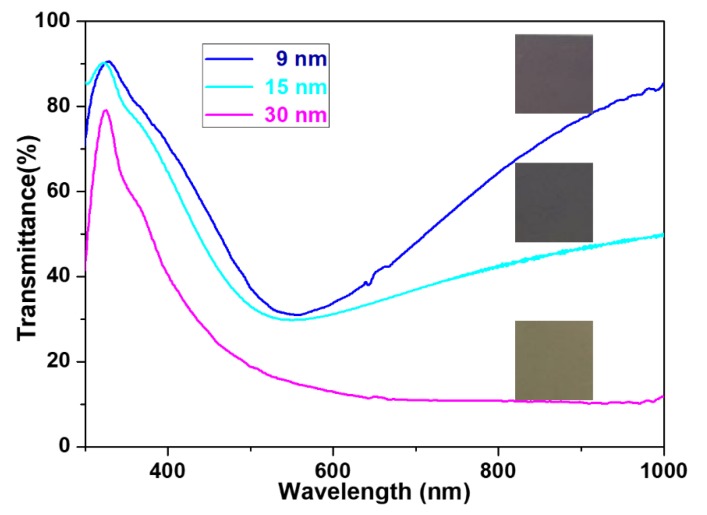
Transmittance of Ag covers immobilized on glass slides with different thicknesses. The squares on the right panel of the figure show photographs of corresponding Ag covers with different thicknesses.

The thickness of Ag cover at Si substrate was evaluated by noncontact mode of AFM and the results are displayed in Figure S5. The transmittance of Ag cover was directly proportional to the thickness. Incident lights with visible and near-infrared wavelengths for surface plasmon resonance excitation will not completely penetrate the Ag cover. Figure 4 displays the UV-vis-NIR transmittance spectra of Ag cover with 9, 15, and 30 nm thickness. Obviously, the 30 nm-thick Ag film (pink trace) showed the lowest transmittance when compared to 9 nm (blue trace) and 15 nm (green trace) thick Ag films, meaning that incident light cannot pass through thick Ag films. The transmittance magnitude of Ag cover films followed the same tendency as transparency observed in SEM images (Figure 3). The digital photographs also presented light absorption properties of films with different thicknesses (squares in Figure 4).

On the other hand, the sandwich nanostructure displayed higher structural stability, which might be linked to three factors. First, the as-synthesized Au nanorings contained an inner Pt skeleton with excellent structural stability against harsh conditions, such as O_2_ plasma treatment, high power light illumination, and extreme pH environment [58]. By contrast, typical pure Au and Ag nanoparticles would fail under harsh conditions because of their relatively weak physical properties, such as lower melting points. Second, illumination of electromagnetic energy can be shunted to both the mirror and cover parts, which would limit damage of nanomaterials through external energy input [15]. Third, the proposed sandwich nanostructure is more closed so that external chemicals cannot easily contaminate the whole nanostructure.

**Figure 5:**
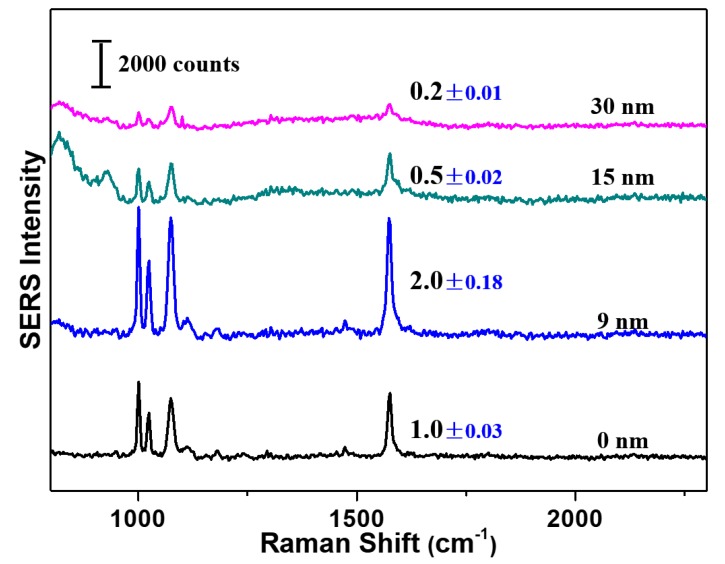
Dependence of surface-enhanced Raman spectroscopy (SERS) intensity on thickness of Ag cover. The relative intensity of each specimen is indicated nearby by the peak at 1573 cm^−1^. The number on the right of each curve shows the thickness of Ag cover.

Before immobilization of Ag cover on monolayer Au nanorings, the benzenethiol was first adsorbed on Au nanorings through immersion in 0.1 M benzenethiol ethanol solution for 1 h. The variation in SERS intensity on the as-fabricated sandwich nanofilms was evaluated by vibration mode of in-plane phenyl ring at 1573 cm^−1^ (Figure 5). It can be seen that SERS intensities changed with thickness of Ag cover. Without the use of Ag cover, SERS of Ag mirror/Au nanorings had intensities normalized as 1.0 (black trace in Figure 5). This SERS signal was attributed not only to Au nanorings but also to Ag mirror-Au nanoring interfacial plasmonic coupling issued from image charge effect oscillation (Figure 2). Using 9 nm Ag cover deposited on monolayer Au nanorings, the SERS intensity increased to 2.0 (blue trace). This value was two-fold higher than that without Ag covers, suggesting the production of additional plasmonic coupling between Au nanorings and Ag covers. As expected, the direct striking of light penetrating Ag cover, as well as image charge at Au nanorings induced by SPR of Ag cover both contributed to enhanced EM field along the junction of Au nanorings and Ag cover. This raised the SERS signal intensity of sandwich-type Ag mirror/Au nanorings/Ag cover film by eight-fold when compared to monolayered Au nanorings deposited on plasmonic inactive Si substrates.

The experimental results indicated that thicker Ag films (15 nm and 30 nm) could not improve SERS intensity anymore. The normalized SERS intensities decreased to 0.5 (green trace) and 0.2 (pink trace) for 15 nm and 30 nm Ag covered sandwiched substrates, respectively (Figure 5). The latter may be ascribed to the ineffective penetration of light through Ag covers, leading to significant decrease in plasmonic coupling of both Au nanorings and junctions between Au nanorings and Ag mirror. A thinner Ag cover might probably provide significantly enhanced SERS signal intensity. However, more investigation dealing with enhancement effect could not be conducted using thinner Ag covers due to their extreme fragile structures.

**Figure 6:**
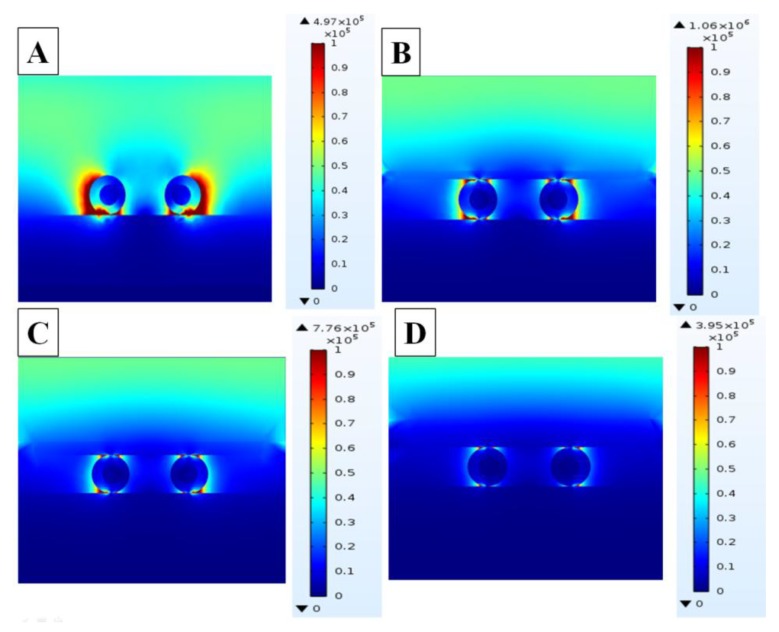
FDTD simulation of electromagnetic field distribution of sandwiched films under the radiation of 633 nm laser: (**A**) Ag mirror/Au nanorings and (**B**–**D**) Ag mirror/Au nanorings/Ag cover with different cover thicknesses of 9 nm, 15 nm, and 30 nm.

To gain a better understanding of SERS behavior as a function of Ag cover thickness, the local electromagnetic field (EM) properties were analyzed using FDTD method. Figure 6 shows the EM field distribution at cross-section of sandwich-type films with different Ag cover thickness subjected to 633 nm laser irradiation. In FDTD simulations, the radii of inner Pt wire and Pt@Au wire were set to 22 nm and 44 nm, respectively. These values were the same as the experimental ones. The maximum EM enhancement (|E|^2^) values for contrast specimens are displayed at the right side of each image in Figure 6. The area of dark red region in Figure 6A for specimens without Ag cover seemed the largest in the contrast specimens, but the maximum EM enhancement of 4.97 × 10^5^ was not the highest, which is lower than the specimen with thin Ag cover of 9 nm in Figure 6B, 1.06 × 10^6^. In other words, the maximum EM enhancement was more critical than the area of electric field distribution. The maximum EM enhancements in specimens with thicker Ag covers of 15 nm and 30 nm were 4.5 × 10^5^ and 3.95 × 10^5^, respectively. It is obvious that the thinner Ag cover, the higher maximum EM enhancement. Due to the technical limitation in cover fabrication, the yield of covers less than 9 nm was low and no thinner Ag covers were evaluated. Consequently, substantially enhanced EM can be achieved with thin Ag cover to allow effective penetration of incident light. A thick Ag cover can block light from penetrating inside, causing inefficient plasmonic coupling among Au nanorings and junction between Au nanorings and Ag mirror. The simulation results showed consistent tendency with the experimental data for SERS signal intensity measurements for Ag covers with various thicknesses.

## 4. Conclusions

A strategy for enhancing SERS intensity was achieved by 3D sandwich nanostructures containing SERS active nanostructure in-between plasmonic mirror and cover films. The Ag mirror/Au nanorings/Ag cover sandwiched nanofilm achieved ~eight-fold higher SERS intensities when compared to films prepared without mirror and cover. The plasmonic resonance coupling along the *z*-axis for Ag mirror/Au nanoring and Au nanoring/Ag cover played a central role in SERS signal enhancement. Although the fabrication complexity makes the present sandwich nanostructure unsuitable for practical use, the significance of this work is to highlight a new dimension to enhance SERS intensity by creating more active sites along the *z*-axis in a 3D sandwich nanostructure for molecular recognition and detection.

## Supplementary Materials

The following are available online at https://www.mdpi.com/2079-4991/10/3/519/s1, Figure S1: SEM images of monolayer Au nanorings without and with Ag cover and their corresponding EDS data, Figure S2: SEM image of Au nanorings self-assembled in LB process using different concentration of MPTMS in the solution, Figure S3: UV-vis-NIR spectrum of the Au nanorings, Figure S4: SERS intensities from blank Ag substrate and monolayer Au nanorings, Figure S5: hFM Height profiles of different thickness of Ag cover films, Equation (S1): wavelength of Raman scattering, and equations for simulation method.
