# Supplementary material for: Synthesis of Monolayer Gold Nanorings Sandwich Film and Its Higher Surface-Enhanced Raman Scattering Intensity
Source: Nanomaterials (Basel). 2020 Mar 13;10(3):519. doi: 10.3390/nano10030519 (PMC7153256; doi:10.3390/nano10030519)
Supplement: Supplementary file 1 [file nanomaterials-10-00519-s001.pdf]

nanomaterials

Supporting Information for

Synthesis of Monolayer Gold Nanorings Sandwich Film and Its Higher Surface-Enhanced Raman Scattering Intensity

Liqiu Zhang [1,2,*] , Tiying Zhu [3], Cheng Yang [3], Ho Young Jang [1], Hee-Jeong Jang [1], Lichun Liu [2] and Sungho Park [1,*]

[1] Department of Chemistry & Department of Energy Science, Sungkyunkwan University, Suwon 440-746, Korea; zeroair@skku.edu (H.Y.J.); snow306@skku.edu (H.-J.J.)
[2] College of Biological, Chemical Sciences and Engineering & Nanotechnology Research Institute, Jiaxing University, Jiaxing 314001, China; lichun.liu@zjxu.edu.cn (L.L.)
[3] School of Physics and Electronics, Shandong Normal University, Jinan 250014, China; 2018020534@stu.sdnu.edu.cn (T.Z.); chengyang@sdnu.edu.cn (C.Y.)
* Correspondence: liqiu0524@zjxu.edu.cn (L.Z.); spark72@skku.edu (S.P.)

Figures, Equation and Simulation:

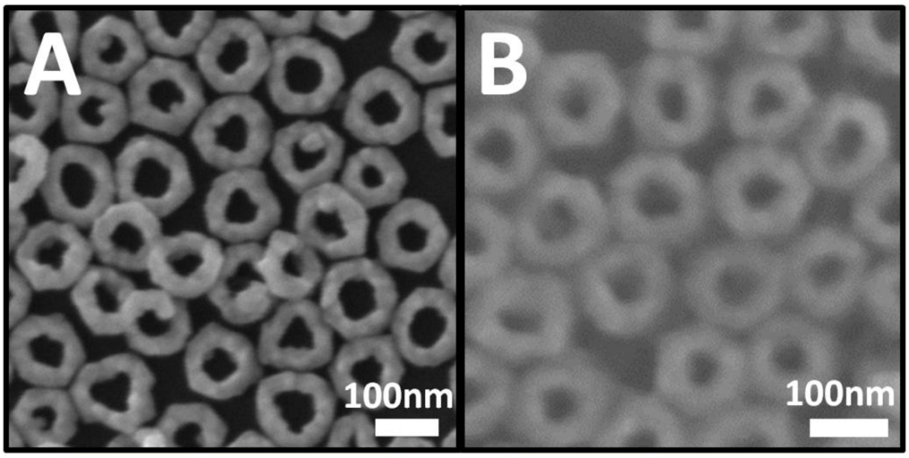

Element	Atomic%
Ag	0.00
Pt	20.08
Au	79.92
Total:	100.00

Element	Atomic%
Ag	3.14
Pt	18.05
Au	78.81
Total:	100.00

Figure S1. Representative SEM images of monolayer Au nanorings without (**A**) and with (**B**) Ag cover and their corresponding EDS data shown below.

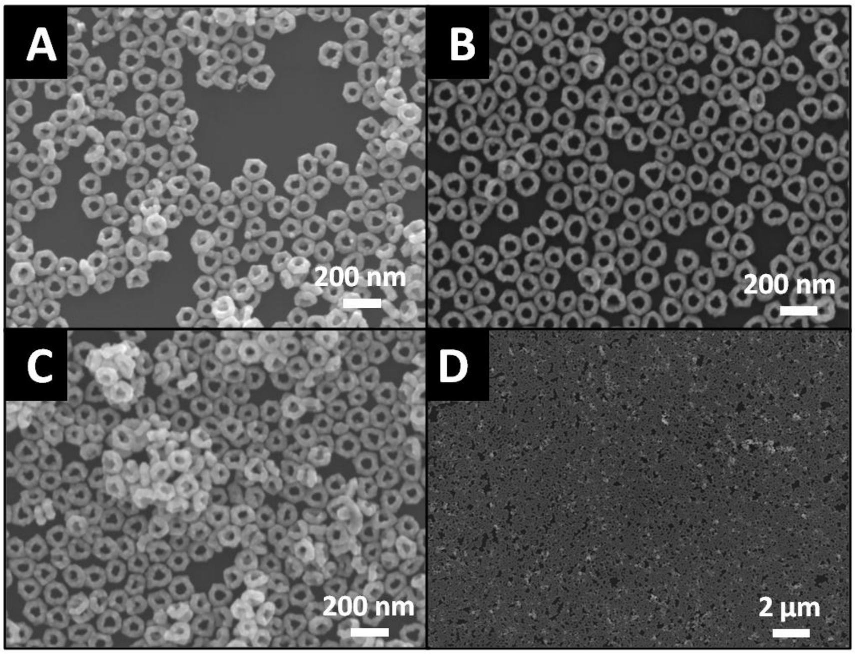

Figure S2. SEM image of Au nanorings self-assembled in LB process using different concentration of MPTMS in the solution: 1 mL 0.01 mM (**A**), 1 mL 1 mM (**B**), 1 mL 10 mM (**C**), (**D**) is the low magnification of (**B**).

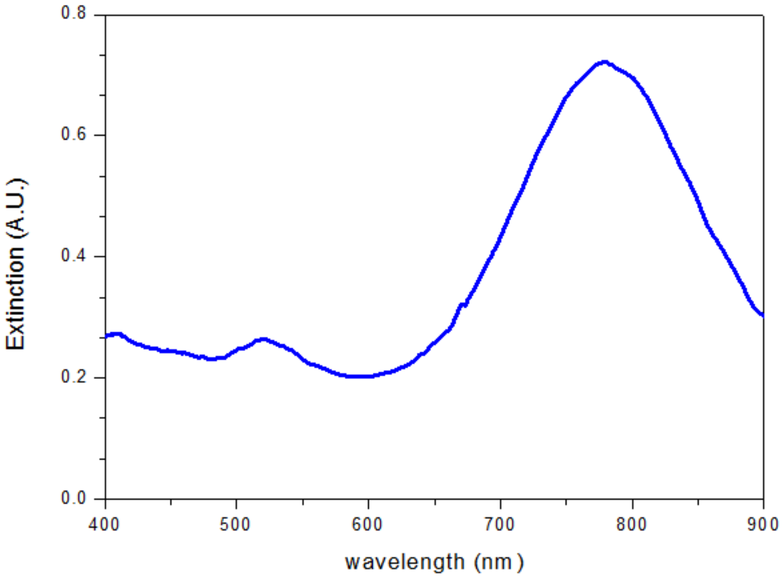

Figure S3. UV-vis-NIR spectrum of the Au nanorings dispersed in deionized water.

Equation S1:

Definition: λ_1, wavelength of incident light; λ_2, wavelength of Raman scattering; $\boldsymbol{\Delta\nu}$, Raman shift.

$$\frac{1}{\lambda_1} - \frac{1}{\lambda_2} = \Delta\nu\ (1000{\sim}1573\ cm^{-1}, see\ Figure\ S4\ )$$

$$\frac{1}{632.8\times 10^{-7}cm} - \frac{1}{\lambda_2} = 1000\sim1573\ cm^{-1}$$

$$\frac{1}{\lambda_2} = 14802\sim14229\ cm^{-1}$$

$$\lambda_2 = 675 \sim 702\ nm$$

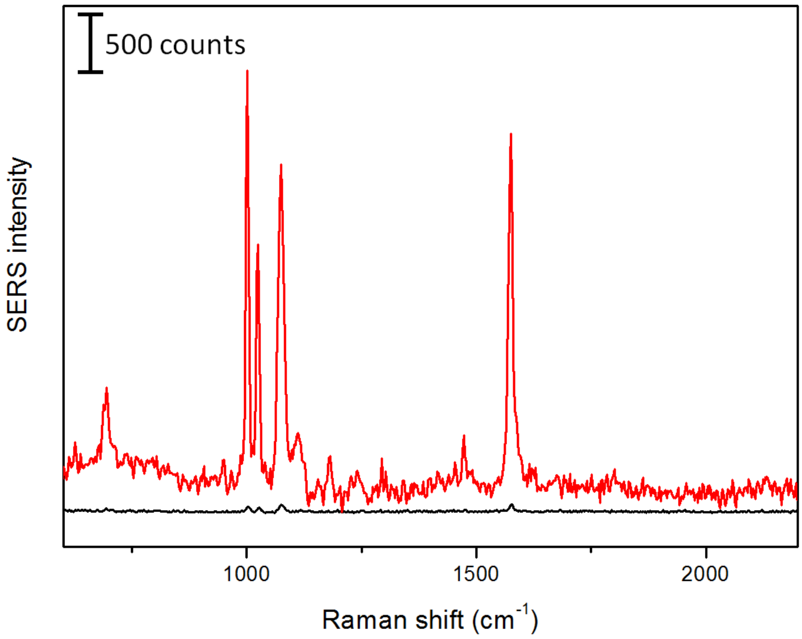

Figure S4 SERS intensities from blank Ag substrate (black), and from monolayer Au nanorings on Ag substrate (red).

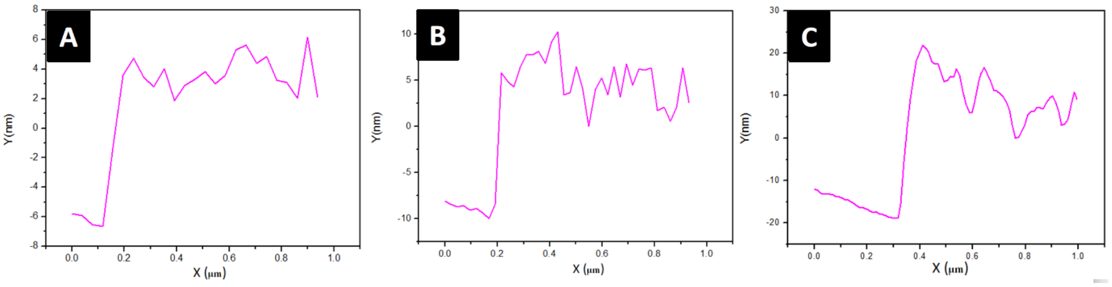

Figure S5. Height profiles of different thickness of Ag cover films, (A) ~9 nm, (B) ~15 nm, and (C) ~30 nm on Si substrate.

Simulation method:

In FDTD simulations, the radii of inner Pt wire and Pt@Au wire were set to 22 nm and 44 nm, respectively. These values were the same as the experimental ones. The EM field distribution at cross-section of sandwich-type films with different Ag cover thickness subjected to 633 nm laser irradiation is simulated. FDTD method is used to solve Maxwell's equations in nonmagnetic materials:

$$\frac{\partial \vec{D}}{\partial t} = \nabla \times \vec{H} \qquad (1)$$

$$\vec{D}(\omega) = \varepsilon_0 \varepsilon_r \vec{E}(\omega) \qquad (2)$$

$$\frac{\partial \vec{H}}{\partial t} = -\frac{1}{\mu_0} \nabla \times \vec{E} \qquad (3)$$

where H, E, and D are the magnetic, electric, and displacement fields, respectively. ε and μ_0 are the complex relative dielectric constant and magnetic permeability coefficient, respectively.

In three dimensions, Maxwell equations have six EM field components. With the assumption that the structure is infinite in the z dimension and that the fields are independent of z, the Maxwell's equations are split into two independent groups of equations that can be solved in the x–y plane only, which results in the transverse electric (TE) and transverse magnetic (TM) equations. Then, we can use the components of E_x, E_y, and H_z to solve TE equations and those of H_x, H_y, and E_z to solve TM equations.

$$\frac{\partial E_z}{\partial y} - \frac{\partial E_y}{\partial z} = -\mu_0 \frac{\partial H_x}{\partial t} \qquad (4)$$

$$\frac{\partial E_x}{\partial z} - \frac{\partial E_z}{\partial x} = -\mu_0 \frac{\partial H_y}{\partial t} \qquad (5)$$

$$\frac{\partial E_y}{\partial x} - \frac{\partial E_x}{\partial y} = -\mu_0 \frac{\partial H_z}{\partial t} \qquad (6)$$

$$\frac{\partial H_z}{\partial y} - \frac{\partial H_y}{\partial z} = \varepsilon \frac{\partial E_x}{\partial t} \qquad (7)$$

$$\frac{\partial H_x}{\partial z} - \frac{\partial H_z}{\partial x} = \varepsilon \frac{\partial E_y}{\partial t} \qquad (8)$$

$$\frac{\partial H_y}{\partial x} - \frac{\partial H_x}{\partial y} = \varepsilon \frac{\partial E_z}{\partial t} \qquad (9)$$

Considering that the function $f(x, y, z, t)$ denotes the electric or magnetic field in the coordinate system, it can be discretized via the central difference approximation in both space and time.

$$\frac{\partial f(x, y, z, t)}{\partial x}\Big|_{x=i\Delta x} \approx \frac{f^n(i+0.5, j, k) - f^n(i-0.5, j, k)}{\Delta x} \qquad (10)$$

$$\frac{\partial f(x, y, z, t)}{\partial y}\Big|_{y=j\Delta y} \approx \frac{f^n(i, j+0.5, k) - f^n(i, j-0.5, k)}{\Delta y} \qquad (11)$$

$$\frac{\partial f(x,y,z,t)}{\partial z}\Big|_{z=k\Delta z} \approx \frac{f^{n}(i,j,k+0.5)-f^{n}(i,j,k-0.5)}{\Delta z} \qquad (12)$$

$$\frac{\partial f(x,y,z,t)}{\partial t}\Big|_{t=n\Delta t} \approx \frac{f^{n+0.5}(i,j,k)-f^{n-0.5}(i,j,k)}{\Delta t} \qquad (13)$$
